# CCR4-NOT Complex 2—A Cofactor in Host Cell for Porcine Epidemic Diarrhea Virus Infection

**DOI:** 10.3390/genes13091504

**Published:** 2022-08-23

**Authors:** Jieru Wang, Hailong Liu, Dongdong Yin, Mei Zhou, Lei Yin, Yuqing Yang, Zishi Guo, Xuehuai Shen, Yin Dai, Shaohua Shi, Shengsong Xie, Ruihong Zhao, Xueli Zhou, Xiaomiao Hu, Hongyan Hou, Chonglong Wang, Xiaocheng Pan

**Affiliations:** 1 Anhui Province Key Laboratory of Livestock and Poultry Product Safety Engineering, Livestock and Poultry Epidemic Diseases Research Center of Anhui Province, Key Laboratory of Pig Molecular Quantitative Genetics of Anhui Academy of Agricultural Sciences, Institute of Animal Husbandry and Veterinary Science, Anhui Academy of Agricultural Sciences, Hefei 230031, China; 2 Key Laboratory of Agricultural Animal Genetics, Breeding and Reproduction of Ministry of Education & Key Lab of Swine Genetics and Breeding of Ministry of Agriculture and Rural Affairs, Huazhong Agricultural University, Wuhan 430070, China

**Keywords:** porcine epidemic diarrhea virus, CNOT2, surface plasmon resonance, interaction

## Abstract

The porcine epidemic diarrhea virus (PEDV) has catastrophic impacts on the global pig industry. However, there is no consensus on the primary receptor associated with the PEDV invasion of host cells. An increasing number of studies have reported that PEDV invading host cells may require collaboration between multiple receptors and to better understand the virus-host interaction during PEDV entry, surface plasmon resonance (SPR) assays are performed to investigate relevant host factors interacting with PEDV spike-1 protein (S1) in Vero and IPEC-J2 cell membranes. Subsequently, the rabbit anti-PEDV S1 polyclonal antibody is used as bait to recognize the complexes of IPEC-J2 membrane proteins with or without PEDV infection, followed by detection using liquid chromatography with tandem mass spectrometry (LC-MS-MS). Our results show that 13 and 10 proteins interacting between the S1 protein and plasma membrane protein of Vero or IPEC-J2 can be identified. More specifically, a total of 11 differentially expressed interacting proteins were identified in IPEC-J2 membrane proteins after PEDV infection, compared to the uninfected group. Furthermore, we found that the differentially interacting protein CCR4-NOT complex 2 (CNOT2), identified in PEDV S1 with plasma membrane proteins of Vero cells, is involved in viral infection. The results show that the knockout of CNOT2 significantly inhibits PEDV replication in vitro. These data provide novel insights into the entry mechanism of PEDV.

## 1. Introduction

The porcine epidemic diarrhea virus (PEDV) causes porcine epidemic diarrhea (PED), a highly contagious disease in pigs characterized by severe enteritis, vomiting, and acute watery diarrhea [1]. PEDV infects pigs at different developmental phases and is associated with 30–80% mortality and up to 100% incidence in suckling piglets [2,3]. PEDV is an enveloped single-stranded RNA virus with a genome length of approximately 28.5 kb. Among the four structural proteins in the genome, the spike (S) glycoprotein (~1383 amino acids) is composed of S1 and S2 proteins. The S1 protein is the PEDV surface antigen regulating the interaction between the host cell receptor protein and the virus and can mediate virus entry [4]. The core mechanism of virus invasion is the binding of the virus to specific receptors on the cell membrane surface. Previous studies indicated that porcine aminopeptidase N (pAPN) is a possible cell surface receptor for PEDV [5,6,7,8]. However, recent studies have reported that the overexpression of pAPN in non-susceptible cells does not induce PEDV infection. The depletion or downregulation of pAPN expression in susceptible cells does not have an inhibitory effect on PEDV infection [8,9,10,11]. Other studies using CRISPR/Cas9-mediated gene editing and somatic cell nuclear transfer demonstrated that pAPN-deficient pigs and mice are susceptible to PEDV infection [5,12,13,14]. Hence, pAPN is a coreceptor assisting PEDV invasion rather than acting as a primary receptor. A recent report indicated that cell surface tight junction proteins, cell adhesion factors, and sugars play a synergistic role as helper molecules of virus invasion and participate in PEDV binding to host cell receptors to trigger endocytosis [15,16,17,18]. For example, sulfated heparin on cell surfaces treated with heparinase could prevent PEDV invasion [15]. Occludin, a tight junction protein, is involved in the internalization process (micropinocytosis) of PEDV infection [16]. The absence of the lymphotoxin β receptor increased the susceptibility to PEDV infection in the porcine small intestinal epithelial cell line (IPEC-J2) by significantly suppressing NF-κB target genes and mucosal barrier integrity-related genes [17]. The extracellular region of transferrin receptor 1 interacts with the PEDV S1 protein to promote PEDV entry via activating the transferrin receptor 1 tyrosine phosphorylation mediated by Src kinase [18]. The primary PEDV receptor remains controversial as PEDV may use more than one receptor to infect host cells. The precise mechanisms regulating PEDV entry into IPEC-J2 and Vero cells remain unknown.

In the current study, we aim to investigate the relevant host factors that directly interact with the PEDV S1 protein to uncover the mechanisms by which virus invasion of the host cell membrane occurs. We identify several proteins interacting between S1 and the plasma membrane protein of Vero or IPEC-J2, respectively. Furthermore, the differentially expressed interacting proteins are also identified in IPEC-J2 membrane proteins after PEDV infection, compared to the uninfected group. Finally, we generate a CNOT2 knockout (KO) Vero cell line by CRISPR/Cas9 technology and observe that CNOT2 KO cells can inhibit PEDV replication. Our study suggests that the CNOT2 protein may interact with PEDV S1 and participate in the replication of PEDV.

## 2. Materials and Methods

### 2.1. Cells and Virus

The porcine small intestinal epithelial cell line (IPEC-J2), Vero, human embryonic kidney cells (HEK 293T), and Cas9 stable expressed Vero cells (Vero-Cas9) were maintained in Dulbecco’s modified Eagle medium (DMEM, Gibco, Grand Island, NE, USA) supplemented with 10% heat-inactivated fetal bovine serum (FBS, Gibco, Grand Island, NE, USA) and antibiotics (100 U/mL of penicillin and 8 µg/mL of streptomycin) in a humidified 5% CO_2_ incubator at 37 °C. A maintenance medium without FBS and supplemented with trypsin (5 µg/mL) (Gibco, Grand Island, NE, USA) was used to prepare virus cultures and virus-infection assays. The PEDV JS-A strain (GenBank: MH748550) was used throughout the current study.

### 2.2. Maltose-Binding Protein (MBP)-S1 Protein Expression and Purification

The PEDV S1 gene was cloned into the pMAL-c5x vector (NEB) for the expression of the PEDV S1-MBP protein. The recombinant plasmid was transformed into *Escherichia coli* BL21 (DE3) and then cultured at 37 °C in Luria–Bertani medium until the OD600 reached 0.6. Subsequently, 0.2 mM of isopropyl β-D-thiogalactoside was added to induce protein expression. After 16 h of incubation, the bacteria were harvested at 18 °C, lysed by ultrasonication, and purified by dextran gel (Sephadex G-100). The purified recombinant protein was stored at –80 °C.

### 2.3. PEDV Infection Assay

IPEC-J2 cells were cultured in a 10 cm cell culture plate and infected with PEDV (MOI = 0.5) at 95% confluence. The infected IPEC-J2 cells were cultured further for 36 h. The morphological changes in PEDV-infected IPEC-J2 cells were observed to determine the sample conditions for surface plasmon resonance (SPR). Then, the cells were rinsed twice in 1× phosphate-buffered saline (PBS) and centrifuged at 1000× *g* for 10 min to collect the cell pellet.

The wide-type (WT) and CNOT knockout (KO) Vero cells were grown to approximately 80–90% confluence in 6-well culture plates and infected with PEDV at 0.01 and 0.1 MOIs, together with 5 μg/mL trypsin (Invitrogen) for 1 h. After washing the unbound virus with PBS, the cells were then cultured in serum-free DMEM with 5 μg/mL trypsin at 37 °C for 24 h. After the observation of the cytopathic effect (CPE), the cells were collected for viral RNA detection by real-time reverse transcription PCR (qRT-PCR), the immunofluorescence assay (IFA), and the median tissue culture infectious dose (TCID50) assay.

For the virtual binding and entry assay, WT and CNOT-KO Vero cells were infected with PEDV at 5 MOI at 4 °C for 1 h (binding), cultured at 37 °C with 5% CO_2_ with 5 μg/mL of trypsin, and then treated at 37 °C for 30 min (entry).

### 2.4. Cell Plasma Membrane Protein Isolation

The plasma membrane proteins of Vero, IPEC-J2, and IPEC-J2 with PEDV infection were isolated from 1.0 × 10^6^ cells using a minute plasma membrane protein isolation and cell fractionation kit (SM-005, Invent Biotechnologies, Plymouth, MN, USA), following the manufacturer’s instructions. The pellets containing plasma membrane proteins were collected to determine the interaction with PEDV by SPR.

### 2.5. Surface Plasmon Resonance (SPR)

In total, two different binding assays were performed on an OpenSPR localized SPR biosensor (Nicoya Lifesciences Inc., Kitchener, ON, Canada), according to the description by McGurn et al. [19]. In the first binding assay, the PEDV S1 protein at a concentration of 50 μg/mL was immobilized on activated COOH-sensor chips serving as the ligand in standard 1-ethyl-3-(3-dimethylpropyl)-carbodiimide plus N-hydroxysuccinimide, which presented strong covalent binding via carboxyl [20]. Subsequently, 30 μg/mL of the plasma membrane protein of Vero or IPEC-J2 was introduced into the sensor chip with PBS as the running buffer. In addition, 50 μg/mL of MBP served as a negative control. The combined compounds could be dissociated from the coating surface by acid and alkali components. Finally, the S1 and plasma membrane proteins were separated by 0.25% SDS in 10 M glycine hydrochloric acid and disrupted the weak protein–protein interaction forces (e.g., hydrogen bond and van der Waals’ forces) [21].

The second binding assay was performed using a rabbit anti-PEDV S1 polyclonal antibody (FriendBio Technology, Wuhan, Hubei, China), used as a bait fixed to the activated COOH-sensor chips. Briefly, the PEDV uninfected IPEC-J2 cells served as controls (group A), while PEDV infected IPEC-J2 cells were the treatment (group B). Membrane proteins with or without PEDV complexes were extracted and introduced into the sensor chip with PBS as the running buffer to bind the S1 antibody. Subsequently, the S1 antibody and the complexes of membrane proteins with or without PEDV infection were separated by 0.25% SDS in 10 M glycine hydrochloric acid.

### 2.6. Liquid Chromatography with Tandem-Mass Spectrometry (LC-MS/MS) Analysis

For the PEDV S1 protein/plasma membrane protein of Vero or IPEC-J2 binding assay, the eluted proteins were separated by electrophoresis on 12% (*w*/*v*) SDS-polyacrylamide gel electrophoresis (SDS-PAGE). A protein band measuring approximately 55 kDa in size was excised from the gels and analyzed further with LC-MS/MS to determine the differentially interacting proteins between the S1 and plasma membrane proteins of Vero or IPEC-J2. In addition, the elution of protein of IPEC-J2, with or without PEDV infection, was directly performed by LC-MS/MS, as previously described [22]. The differential proteins’ collective peptide mass fingerprinting and LC-MS/MS inquiries used the AB SCIEX Triple TOF™ 5600 plus mass spectrometer coupled to the Protein-pilot v4.0 software from the NCBI database (Taxonomy: NCBI txid60711 and NCBI txid9823). We selected the SWISSPROT *Chlorocebus sabaeus* (Vero cells) and *Sus scrofa* (IPEC-J2 cells) databases using the following default parameters: a peptide tolerance of 1.0 Da and specificity of one missed cleavage; carbamidomethyl modification of cysteine; and acetylation of the NH2 terminal ends of lysine. Proteins with correlated *p*-values < 0.05 were considered as statistically significant categories and were conducted using the default settings. The main functions of each protein was confirmed via the UniProt (https://www.uniprot.org/, accessed on 17 June 2022) hyperlink embedded in each node.

### 2.7. Generation of CNOT2 Knockout Cell Lines

To generate CNOT2 (ENSCSAG00000004177) knockout Vero cell lines, sgRNAs were designed using sgRNAcas9-AI (http://123.57.239.141:8080/home, accessed on 10 February 2021) and cloned into linearized lenti-sgRNA-EGFP to produce recombinant lentivirus packaging puromycin resistance (psPAX2, Addgene12260, pMD2.G, Addgene12259), as previously described [23]. The sequences of CNOT2 sgRNA are CNOT2-sgRNA-F: caccgGTGCACTAGGCCTTCCAATG and CNOT2-sgRNA-R: aaacCATTGGAAGGCCTAGTGCACc. Then, the CNOT2 sgRNA lentivirus was transduced into Vero-cas9 cells. On the third day, following transduction, cells with GFP expressions were enriched by fluorescence-activated cell sorting. For the analysis, genomic DNA was extracted (TIANamp Genomic DNA Kit, Tiangen) and PCR amplified. The CNOT2 knockout was confirmed by sequencing the PCR products (Microsynth Seqlab, Göttingen, Germany) with the following primers: CNOT2-seq-F: TGCTGGCATTTTATGTTGGGG and CNOT2-seq-R: TCAGTGAAAGGCACTGAACCA. Lack of protein expression was confirmed by western blot for CNOT2.

### 2.8. Cell Counting Kit-8 Assay

The cell viability was measured by the Cell Counting kit-8 (CCK8) (Mei5bio, Beijing, China) assay following the instructions mentioned. The CNOT2 -KO and WT Vero cells were seeded to 96-well plates and infected with or without PEDV for 1 h at 0.01 MOI. Subsequently, the cells were washed three times with PBS and incubated for 36 h, then the cultured supernatant in each well was replaced with 110 µL DMEM containing 9.09% CCK-8 reagent, followed by culturing in the dark for 4 h. After gentle shaking, the optical density 450 (OD450) was measured by a microplate reader. Cell viability value was calculated according to the equation: the cell viability value = (As − Ab) ÷ (Ac − Ab) × 100%, where, As: after incubation with CCK-8 solution, the OD450 values of PEDV infection; Ab: after incubation with CCK-8 solution, the OD450 values of PEDV infection wells without cells; Ac: after incubation with CCK-8 solution, the OD450 values of un-infection wells.

### 2.9. Absolute Real-Time Reverse Transcription PCR (qRT-PCR) Analysis

Viral RNA from cell suspensions was extracted with a viral RNA extraction kit (#9766, TaKaRa, Shanghai, China) according to the instructions. The complementary DNAs (cDNA) were obtained by RT-PCR with a PrimeScript™ RT reagent Kit with a gDNA Eraser (TaKaRa, Shanghai, China). The absolute qRT-PCR for quantifying the PEDV genome was prepared with the RealUniversal SYBR Green Premix (Tiangen, Beijing, China) and The PEDV a 186-fragment gene was cloned into the pMD19-T vector and used as an internal reference for the quantification of PEDV copy numbers (copies/ul = 6.02 × 10^23^ × plasmids concentrations/(DNA Length × 660). Using the standard plasmid as a template, the qRT-PCR amplification after doubling dilution obtained the correlation between the Ct value and the virus copy number, and the standard curve y = −3.2511x + 39.778 (R^2^ = 0.9632), where y is the Ct value and x is Log_10_ (copy number). The primers sequences for a 186-fragment gene: PEDV-186-F: 5′-TACTAAGCGTAACATCCTGCC-3′; PEDV-186-R: 5′-GTAGTACCAATAACAACCGAAGC-3′.

### 2.10. Tissue Culture Infectious Dose 50 (TCID50) Assay

Virus stock solutions were serially diluted before they were inoculated on the confluent Vero cell monolayers grown in the 96-well plates, followed by washing 3 times with PBS. A total of 8 wells were inoculated with 100µL at each dilution, and the plates were incubated at 37 °C with 5% CO_2_ for 2 days. Wells with a syncytium formation, the specific cytopathic effect caused by JS-A, were classified as PEDV positive. PEDV titration was calculated by TCID_50_ following the Reed–Muench method established by L. J. Reed and H. Muench [24].

### 2.11. Immunofluorescence Assay (IFA)

The CNOT2 -KO and WT Vero cells infected with PEDV at different MOIs were seeded onto 6-well plates, then fixed with 4% paraformaldehyde for 20 min at room temperature (RT) and permeabilized with 0.3% ice-cold TritonX-100 in PBS for 10 min at RT. After being washed three times with PBS, the cells were blocked in PBS containing 1% bovine serum albumin (BSA) for 30 min at room temperature. The cells were incubated with anti-PEDV N-protein antibodies in PBS for 1 h at 37 °C, followed by 3 washes with PBS. Subsequently, the cells were incubated with Alexa 488-labeled anti-mouse antibody (Antgene, Wuhan, China) for 45 min at 37 °C. After 3 washes, the cells were treated with DAPI dihydrochloride at RT for 10 min in the dark (Beyotime Biotechnol, Shanghai, China) to stain the nuclei.

### 2.12. Statistical Analysis

The values are expressed as the mean ± SD. Statistical analyses were performed using GraphPad 9 software (San Diego, CA, USA). An unpaired *t*-test was performed to investigate the differences between the control and treatment groups (ns = no significance; * *p* < 0.05; ** *p* < 0.01; *** *p* < 0.001).

## 3. Results

### 3.1. The Interacting Proteins between PEDV S1 and the Plasma Membrane Protein of Vero and IPEC-J2 Cells

**Figure 1:**
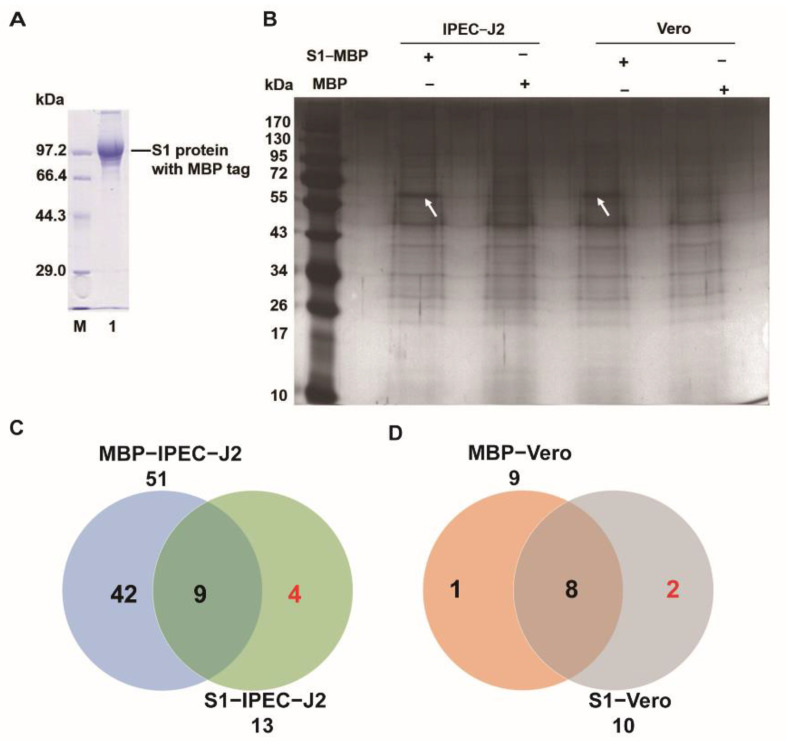
Analysis of the interaction between PEDV S1 and the plasma membrane protein of Vero or IPEC-J2 by SPR assay. (**A**) SDS-PAGE (12% *w*/*v*) is used to determine the expression of the recombinant PEDV S1 protein. Lane M: PageRuler Pre-stained Protein Ladder; lane 1: purified S1 protein with MBP tag. (**B**) The interaction between PEDV S1 and the plasma membrane protein of Vero or IPEC-J2 is measured by an SPR assay. The eluted proteins are separated by electrophoresis on 12% gels with silver staining. The arrow indicates the differentially interacting proteins between S1-MBP and plasma membrane protein of Vero or IPEC-J2 compared to the control group (MBP/plasma membrane protein of Vero or IPEC-J2). (**C**,**D**) Venn diagram of the interacting proteins in 55 kDa protein band by LC-MS/MS analysis. The total numbers of plasma membrane proteins of Vero or IPEC-J2 interacting with S1 protein or MBP-tag are labeled below S1 protein or MBP tag.

To explore the interacting proteins between PEDV S1 and the plasma membrane protein of Vero and IPEC-J2, the *S1* coding sequence of the PEDV JS-A strain was selected for the synthesis. Subsequently, the expression of the PEDV S1 protein was detected in the specific band of ~85.5 kDa (Figure 1A), including an MBP tag that was approximately 42.5 kDa in length. Furthermore, the binding ability between PEDV S1 and plasma membrane protein of Vero and IPEC-J2 was measured by the SPR assay. The result shows that a protein band measuring approximately 55 kDa in size can be observed only in the S1 and plasma membrane proteins of the Vero or IPEC-J2 groups (Figure 1B). Subsequently, the 55 kDa protein band represented the interactional proteins of the plasma membrane protein of Vero or IPEC-J2 with PEDV S1-MBP, compared to the interactional proteins with the MBP tag and was excised from the gels to identify the protein components with LC-MS/MS.

In total, we identified 13 (9 plus 4) and 10 (8 plus 2) proteins interacting between S1-MBP and the plasma membrane protein of Vero or IPEC-J2, respectively (Figure 1C,D). Finally, we identified six differentially interacting proteins by LC-MS/MS. Four proteins (ATPA1, HSP90AA1, KRT80, and VIM) of *Sus scrofa* and two proteins (CNOT2 and Annexin) belonging to *Chlorocebus sabaeus* were listed in Table 1 and revealed the differentially interacting proteins between S1 and the plasma membrane protein of IPEC-J2 and Vero.

### 3.2. Expression Analysis and Screening Proteins Associated with PEDV Entry

Table 2. we extracted membrane proteins of IPEC-J2-infected PEDV and used an S1 rabbit polyclonal antibody as bait to capture the membrane proteins in an indirect SPR assay. We observed that a total of 11 and 7 membrane proteins were fished by the PEDV S1 rabbit polyclonal antibody that recognized the complexes of IPEC-J2 and Vero cell membrane proteins with or without PEDV infection by LC-MS/MS, respectively (Table 2). We found that the membrane proteins fished in the two assays are completely different. However, among these differential expression proteins, CNOT2 was identified both in the two SPRs.

### 3.3. CNOT2 Knockout Inhibits PEDV Infection in Vero Cells

**Figure 2:**
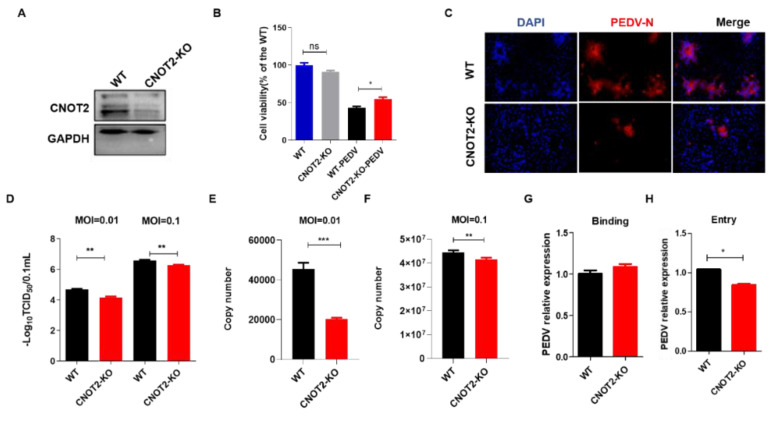
Effect of CNOT2 on PEDV infection of Vero cells. (**A**) Western blot was performed in CNOT2 KO and WT Vero cells. (**B**) Cell viability was evaluated by CCK-8 assay. All the values were normalized to the WT group, representing 100% cell viability. WT and CNOT2-KO cells were infected or uninfected with PEDV for 36 hpi at 0.01 MOI. (**C**) WT and CNOT2 KO cells were infected with 0.01 MOI PEDV at 36 hpi for immunofluorescence analysis with PEDV-N. (**D**–**F**) WT and CNOT2 KO cells infected with PEDV (0.01 and 0.1) at 36 hpi; whole cell lysates (WCLs) and RNA harvested TCID50 (**D**) and the absolute quantification qRT-PCR (**E**,**F**), respectively. (**G**,**H**) WT and CNOT2-KO infected with PEDV at MOI of 5 for 1 h at 4 °C (binding) and warmed to 37 °C for 30 min to initiate entry. The binding and entry of PEDV were quantitated by RT-qPCR; viral RNA was harvested for absolute qRT-PCR. All of the experiments were independently performed three times. Data are represented as means ± S.D.; ns: not significant. *p*-values are determined by two-sided Student’ s *t*-test, * *p* < 0.05; ** *p* < 0.01; *** *p* < 0.001.

We constructed the CNOT2 knockout cells by introducing sgRNAs into Vero-Cas9 cells and compared them to cells transduced with an empty lenti-sgRNA-EGFP vector. The inactivation of CNOT2 in clone KO was confirmed by western blot. We observed that the protein expression of CNOT2 was significantly downregulated in CNOT2-KO compared to WT cells (Figure 2A). To evaluate the effect of CNOT2 on cell proliferation, cell viability was measured using the Cell Counting kit-8 (CCK8) assay. The value of each group was normalized by the average value (set at 100%) of the WT group. The results of the CCK8 assay showed no difference in CNOT2-KO and WT cells. However, the cell viability value was increased after PEDV-infected in CNOT2-KO compared with WT cells (Figure 2B). To further estimate the role of CNOT2 in PEDV infection, immunofluorescence analysis was performed based on PEDV N-protein expression at 0.01 MOI 36 h post-infection (hpi). The result showed that the expression of the PEDV-N protein was significantly inhibited in CNOT2 KO cells (Figure 2C). In addition, viral concentrations of WT and CNOT2-KO cells at different MOIs (0.01 and 0.1) with 36 hpi were performed by TCID_50_ and absolute quantification qRT-PCR of the PEDV genome copy number, and we observed that the viral loads were significantly reduced in PEDV replications at different MOIs, and the knockout of the CNOT2 gene significantly inhibited PEDV replication (Figure 2D–F). Subsequently, we performed a qPCR analysis to investigate the effect of CNOT2 regulation on PEDV binding and entry. The results show that PEDV entry is significantly decreased in CNOT2 KO cells than in WT cells (Figure 2G,H). Therefore, these results showed that the knockout of CNOT2 significantly inhibits PEDV replication.

## 4. Discussion

The first step in virus invasion is receptor binding on the surface of the cell membrane. However, the entry mechanism of PEDV remains largely unclear. An increasing number of studies have reported that PEDV invading host cells may require collaboration between multiple receptors. We investigated the factors critical to PEDV entry using two different surface plasmon resonance (SPR) assays based on the direct and indirect interactions between the PEDV S1 protein and IPEC-J2/Vero cell membrane proteins. Complexes of the IPEC-J2 membrane proteins with the PEDV virion were detected using a rabbit anti-PEDV S1 polyclonal antibody. Notably, CNOT2 was identified in the interaction with PEDV S1 protein, with preliminary tests showing PEDV resistance in CNOT2 KO Vero cells.

This study utilized the SPR approach to examine CNOT2 in PEDV infection and preliminarily reports, for the first time, showing that CNOT2 interacts with the PEDV S1 protein and plays a positive role in resistance activity against PEDV, which might serve as a potential target for restricting PEDV infection. CNOT2 regulates the deadenylase activity and structural integrity of the CCR4 NOT complex. Furthermore, CNOT2 supports Dengue virus infection by inhibiting JAK-STAT antiviral signaling via its interaction with CNOT6/6L and CNOT7/8 deadenylases [25]. Moreover, several studies showed that apoptosis [26] and autophagy associated with p62/SQSTM1 degradation [27] are enhanced in CNOT2-depleted cells. However, the biological function of CNOT2 in PEDV infection is poorly understood. Although the knockout of CNOT2 slightly reduces cell growth, the increase in the cell viability value and decrease in PEDV invasion after PEDV-infected in CNOT2-KO compared with WT cells. Hence, we hypothesize that CNOT2-KO cells possess resistance activity against PEDV via suppressing PEDV invasion rather than inducing apoptosis. On the other hand, we found the effect of CNOT2 knockdown on PEDV is moderate in high MOI (0.1 and 5). However, the expression of the PEDV-N protein and viral load are significantly inhibited in CNOT2 KO cells at a lower MOI (0.01) in Figure 2C–F, which suggests that CNOT2 is a cofactor for PEDV invasion, and a collaboration between multiple receptors is required for PEDV invading host cells.

A growing number of studies reported that integrins are involved in *CoV* entry [28]. Integrins are cell surface αβ-heterodimeric glycoproteins that contribute to diverse cellular functions and act as receptors or co-receptors for viral infection [29]. Integrin αvβ3 enhances PEDV replication in Vero E6 and porcine intestinal epithelial cells [30]. Chen et al. reported that the PEDV virus was transferred to intestinal epithelial cells via cell-to-cell contact by blood-derived CD3+ T cells in neonatal piglets via blood circulation following an increase in gut-homing integrin α4β7 [31]. The results illustrate that integrins α3 and β are involved in PEDV infection. However, the precise underlying mechanism is not fully understood. Future experiments should investigate this phenomenon in more detail using pull-down and co-immunoprecipitation assays.

We also observed that the cytoskeleton protein, vimentin, was involved in the interaction between the purified PEDV S1 protein and the plasma membrane proteins of Vero and IPEC-J2 via SPR assays. However, although the prokaryotic-purified S1 protein was co-expressed with the MBP tag, which promotes protein solubility and correct folding, the low-level modification, non-natural fold, and higher molecular weight may have affected the structure and function of the target protein. Hence, the membrane proteins of IPEC-J2 infected with PEDV were extracted and fished by using the rabbit anti-PEDV S1 polyclonal antibody in the second SPR assay, which also appeared as tremendous background noise by non-specific binding. Coincidentally, vimentin was only detected by LC-MS/MS in the S1/IPEC-J2 group. However, the gray level of the 55 KDa band indicated by the two arrows is similar in Figure 1A,B. In addition, the number of identified proteins associated with PEDV S1 and plasma membrane proteins of Vero were far fewer than the proteins associated with IPEC-J2 based on the Veen diagram (Figure 1C,D) and Table 1. Hence, we may lose some import proteins (e.g., vimentin) associated with PEDV S1 and the plasma membrane proteins of Vero.

It was reported that vimentin acts synergistically as a receptor, together with ACE2, of SARS-CoV and participates in virus invasion [32] and interacts with transmissible gastroenteritis virus (TGEV) N protein to participate in virus replication [33]. But vimentin has not been previously reported in PEDV infection. In this study, vimentin was identified in two separate SPR methods, suggesting the importance of determining the mechanisms of vimentin involved in PEDV entry, and further investigations of the regulatory mechanism of vimentin in PEDV entry are warranted.

## Figures and Tables

**Table 1:** Characteristics of differentially interacting proteins identified in PEDV S1 with the plasma membrane protein of Vero or IPEC-J2.

Protein ID	Names	Major Protein Function	Coverage (%)	Length	Mass (Da)	Unique Peptide	Identified by
tr|I7HD36 _PIG	Sodium/potassium-transporting ATPase subunit α (ATPA1)	ATP-binding, ATP hydrolysis activity	6.86	1021	112,679.8	6	S1/IPEC-J2
tr|P79326_PIG	Heat-shock protein 90 protein-α (HSP90AA1)	Unfolded protein binding, ATP-binding	3.42	789	90,639.5c	1	S1/IPEC-J2
tr| A0A287A2M2_PIG	Keratin 80 (KRT80)	Intermediate filament	2.62	433	48,451.4	2	S1/IPEC-J2
sp|P02543| VIME_PIG	Vimentin (VIM)	Intermediate filament	12.86	466	53,667.2	7	S1/IPEC-J2
tr| A0A0D9QVZ5_CHLSB	CCR4-NOT transcription complex subunit 2 (CNOT2)	Positive regulation of cytoplasmic mRNA processing body assembly	0.66	540	59,738	2	S1/Vero
tr| A0A0D9SDK6 _CHLSB	Annexin	Calcium ion binding, cytoskeletal protein binding	2.95	339	38,744.8	1	S1/Vero

Abbreviations: Coverage (%) is the coverage of the identified peptide of the protein and confidence interval ≥ 95%; a unique peptide is defined as a peptide, irrespective of its length, which exists only in one protein of a proteome of interest. The main functions of each protein are reported in the UniProt database (https://www.uniprot.org/, accessed on 17 June 2022).

**Table 2:** Characteristics of differentially interacting proteins with plasma membrane proteins of IPEC-J2 after PEDV infection, as identified by the PEDV S1 polyclonal antibody.

**Group A**	**No.**	**Protein ID**	**Names**	**Major Protein Function**	**Abbreviation**	**Coverage (%)**	**Length**	**Mass**	**#Unique Peptide**
	1	tr|F1SGG3_PIG	Keratin 1	Intermediate filament	KRT1	9.221	624	64,874	6
	2	tr|A5A759_PIG	Keratin 2A	Intermediate filament	KRT2A	5.321	639	65,663	3
	3	tr|F1SGG6_PIG	Keratin 5	Intermediate filament	KRT5	3.299	576	61,358.9	2
	5	tr|A0A287BSX6_PIG	Keratin 14	Intermediate filament	KRT14	5.765	503	54,723.5	2
	4	tr|A0A287B4G4_PIG	Rac family small GTPase 1	Regulation of actin cytoskeleton organization	RAC1	9.38	192	21,449.9	2
	6	tr|A0A287A2M2_PIG	Keratin 80	Intermediate filament	KRT80	2.309	433	48,451.4	1
	7	tr|Q0PY11_PIG	Elongation factor 1-α	Promoting the GTP-dependent binding of aminoacyl-tRNA to the A-site of ribosomes during protein biosynthesis	EEF1A	15.25	59	6246.3	1
**Group B**	**No.**	**Protein ID**	**Names**	**Major Protein Function**	**Abbreviation**	**Coverage (%)**	**Length**	**Mass**	**#Unique Peptide**
	1	sp|P02543|VIME_PIG	Vimentin	Intermediate filament	VIM	17.6	466	53,667.2	7
	2	tr|F1RT622_PIG	Integrin subunit α 3	Virus receptor	ITGA3	2.12	1037	115,677.5	2
	3	tr| K7GS94_PIG	Integrin β	Virus receptor	ITGB1	1.25	853	93,820	1
	4	tr|A0A287AY77_PIG	CCR4-NOT transcription complex subunit 2	Positive regulation of cytoplasmic mRNA processing body assembly	CNOT2	1.98	540	59,738	1
	5	tr|B2CZF5_PIG	Monocarboxylic acid transporter 1	Proton-coupled monocarboxylate transporter	SMCT1	2.59	501	54,246.8	1
	6	tr|K7GSD2_PIG	Guanine nucleotide-binding protein G(s) subunit α	G-protein β/γ-subunit complex binding	GNAS	1.74	632	69,389.4	1
	7	tr|F2Y8C4_PIG	G protein-coupled receptor family C group 5 member C	G protein-coupled receptor activity	GPRC5C	3.46	434	46,933.2	1
	8	tr|F2Z565_PIG	Solute carrier family 25	Catalyzes the exchange of ADP and ATP across the membrane	SLC25A5	6.38	298	32,955	1
	9	tr|I3LB80_PIG	Solute carrier family 3 member 2	L-alanine transmembrane transporter activity	SLC3A2	1.76	568	61,613.4	1
	10	tr|K7GKA5_PIG	Magnesium transporter 1	Protein modification, protein glycosylation	MAGT1	2.419	372	42,330.2	1
	11	tr|A0A286ZLL3_PIG	Receptor of activated protein C kinase 1	Ribosome binding	RACK1	3.623	276	30,505.2	1

Abbreviations: Group A represents the proteins identified in the complexes of membrane proteins without PEDV infection after separation from the S1 antibody. Group B represents the proteins identified in the complexes of membrane proteins with PEDV infection after separation from the S1 antibody. The main functions of each protein are reported in the UniProt database (https://www.uniprot.org/, accessed on 17 June 2022).
